# Population structure of Mycobacterium avium subsp. hominissuis provides new insights into genomic evolution

**DOI:** 10.1099/mgen.0.001543

**Published:** 2025-11-04

**Authors:** Idowu B. Olawoye, David Alexander, Jennifer L. Guthrie

**Affiliations:** 1 Department of Microbiology and Immunology, University of Western Ontario, London, ON, Canada; 2 Cadham Provincial Laboratory, Diagnostic Services, Shared Health, Winnipeg, MB, Canada; 3 Department of Medical Microbiology and Infectious Diseases, University of Manitoba, Winnipeg, MB, Canada; 4 Public Health Ontario, Toronto, ON, Canada

**Keywords:** genetic recombination, genomics, *Mycobacterium avium* subsp. *hominissuis* (MAH), One Health, phylogenetics, population structure

## Abstract

*Mycobacterium avium* subsp. *hominissuis* (MAH) is a clinically important species of non-tuberculous mycobacteria that causes infections in a variety of hosts. This opportunistic pathogen is widespread in the environment, including natural and engineered water systems across the globe. To examine the current genetic diversity of this organism, we analysed 702 MAH genomes isolated from humans, pigs and environmental sources across six countries and three continents. Through Bayesian population structure analysis, we identified ten distinct global lineages, including seven previously described and three new ones. Several lineages exhibited regional clustering, including sequence cluster 6 (SC6) in Germany and SC8 in both the USA and Germany. Further analysis identified recombination hotspots in membrane proteins associated with virulence, antimicrobial resistance and immune modulation, driven by insertion sequences and other elements that frequently integrate at tRNA gene sites. This work demonstrates the remarkable genomic diversity of MAH and provides insight into the evolutionary mechanisms that contribute to its success as a pathogen in both humans and animals.

**Impact Statement**

*Mycobacterium avium* subsp. *hominissuis* (MAH) is an opportunistic, environmentally acquired pathogen in both humans and animals. Using Bayesian population structure analysis, we have provided an updated global lineage schema for these genetically diverse bacteria using 3,976 core genes. Through analysis of 702 genomes, we were able to identify genes under selective pressure in different environmental niches that reflect the evolutionary patterns of the bacteria, allowing survival and adaptation to environmental stress. This study adds to the body of knowledge by identifying key genes that are associated with virulence and antimicrobial resistance in MAH, contributing to One Health approaches to addressing antimicrobial resistance.

## Data Summary

All raw sequencing data analysed in this study were obtained from publicly available datasets in the NCBI Sequence Read Archive (SRA). No new sequencing data were generated, and the relevant BioProject accessions are listed in the Methods section. The data and code used to generate the figures are publicly available on GitHub (https://github.com/guthrielab/MAH_Pop_Structure_paper).

## Introduction

*Mycobacterium avium* is a highly diverse species of non-tuberculous mycobacteria (NTM). Originally identified as the causative agent of tuberculosis in birds, *M. avium* is now recognized as an opportunistic pathogen capable of infecting a wide range of hosts. *M. avium* subsp. *paratuberculosis* (MAP) is responsible for Johne’s disease, a chronic enteric infection most commonly affecting ruminants, such as cattle and goats [1,2]. In contrast, *M. avium* subsp. *hominissuis* (MAH) is most often detected in pigs and humans [3,5]. This ubiquitous subspecies has also been recovered from soil, dust and household water sources, including those of individuals with MAH-related pulmonary infections. These findings suggest that both humans and animals may acquire MAH through environmental exposure to contaminated water or soil [6,7].

 *M. avium* strains exhibit considerable phenotypic and genetic diversity, likely due to their adaptation to complex environmental niches, which provide opportunities for horizontal gene transfer and recombination [8]. Molecular methods such as insertion sequence RFLP, variable number tandem repeat analysis and targeted sequencing of 16S rRNA, *hsp65*, *rpoB* and the 16S–23S internal transcribed spacer (ITS) region have been used to study genetic variation in MAH [9,11]. While these approaches have allowed some classification of isolates, they often produce pseudo-clustering of unrelated strains [12,15]. Furthermore, reliance on conserved genetic regions fails to capture the full breadth of the ~5 Mbp MAH genome, limiting the ability to resolve phylogenetic relationships, track transmission and detect lineage-specific traits.

 Whole-genome approaches overcome these limitations by enabling high-resolution analysis of population structure and recombination across the entire core genome. For example, a study conducted in Japan used 36 global isolates to investigate population structure and lineage-specific genes, identifying five MAH lineages, including two restricted to East Asia, referred to as MahEastAsia1 (MahEA1) and MahEastAsia2 (MahEA2) [12]. Similar patterns of phylogeographic structuring have been observed in other mycobacteria, such as *Mycobacterium tuberculosis*, where distinct lineages are associated with specific human populations and geographic regions [16]. More recent studies that incorporated broader global sampling from both clinical and environmental sources revealed two additional MAH lineages, referred to as sequence cluster 4 (SC4) and SC5 [14,17]. It is hypothesized that some of these lineages emerged through recombination between existing MAH lineages and other environmental mycobacterial species, with recombination hotspots frequently involving genes associated with cell surface structures [12,17].

 With the increasing availability of whole-genome sequencing data, this study builds upon previous efforts to characterize MAH lineages by incorporating a broader set of genomes from diverse geographic regions and host sources. We aimed to update and refine the population structure, investigate the phylogeographic distribution and examine recombination dynamics among lineages. Furthermore, our study attempts to investigate the extent and nature of homologous recombination, including the identification of genomic regions contributing to lineage diversification. A deeper understanding of these processes can support improved transmission tracking, inform public health responses and guide future research on virulence and antimicrobial resistance within a One Health framework. By leveraging publicly available sequence data, our study provides a more comprehensive view of the evolutionary relationships and genetic complexity of this heterogeneous subspecies.

## Methods

### Data acquisition

 This study analysed publicly available MAH genomes that were sequenced using Illumina paired-end technology. The raw sequencing data of 726 MAH genomes were retrieved from the National Center for Biotechnology Information (NCBI) Sequence Read Archive (SRA) using ‘*Mycobacterium avium* subsp. *hominissuis*’ as the keyword for searching in the SRA database, with BioProject included as a filter, and further selecting genomes that have ‘*Mycobacterium avium* subsp. *hominissuis*’ indicated as ‘organism’ in the metadata. The selected genomes originated from the following BioProjects: PRJNA734948, PRJDB11434, PRJNA319839, PRJNA315990, PRJEB70863, PRJEB57933, PRJNA339271 and PRJNA576232 [7,17,21]. As reference representatives of previously described MAH lineages, we included five genome assemblies (A5, 2495, OCU491, OCU464 and OCU462) corresponding to SCs or lineages SC1, SC3, SC4, MahEA1 and MahEA2, respectively [12,14, 17]. For sequence information see Table S1, available in the online Supplementary Material.

### Genome assembly, annotation and pan-genome analysis

 Illumina sequencing reads were processed using the Bactopia [18] v3.1.0 pipeline, which uses FastQC [19] and fastp [20] for quality checking and trimming. *De novo* genome assembly was achieved with Shovill [21]. Average nucleotide identity (ANI) was calculated for all genome pairs using fastANI [22]. Genomes with ANI values below the 95% threshold, as well as those with total contig sizes less than 4,000,000 bp or greater than 7,000,000 bp, were excluded. Following quality control, 702 of the 726 genomes were retained for downstream analysis. Genome annotation was conducted with Prokka [23].

 Pan-genome inference was conducted with Panaroo [24] using the *--clean-mode strict* parameter and a core gene threshold value of 95%. Phylogenetic analysis was based on the alignment of 3,976 core genes shared by at least 95% of the isolates and constructed using IQ-TREE [25]. The maximum-likelihood tree was built using the HKY model and 1,000 ultrafast bootstrap replicates. The resulting Newick file was processed and visualized using the treeio and ggtree packages in R v4.2 [26,27]. Plasmids were identified and reconstructed with MOB-suite [28], and all identified plasmids per genome were counted, while the mean and sd were computed in R v4.2.

### Population structure and recombination analysis

 MAH lineages were inferred using rhierBAPS [29], an R implementation of the hierarchical and spatially Bayesian population clustering algorithm hierBAPS [30]. Core-genome alignments of all 702 isolates were analysed with parameters set to *max.depth=2* and *n.pops=20*. Lineages were defined based on level 1 of the rhierBAPS partition output.

 To assess recombination between lineages, we analysed the core-genome alignment and rhierBAPS-defined lineage assignments using fastGEAR [31]. Due to the high computational demands of analysing all 702 genomes, we randomly subsampled 100 genomes (~10 per lineage) using the *sample* function in R. Recombination analysis was conducted with fastGEAR’s default parameters, which include 15 learning iterations for convergence. This tool identifies both ancestral and recent recombination events between lineages.

 Recent recombination events were visualized based on the most recent common ancestor, allowing us to determine the genomic segments acquired from donor lineages and their relative contributions. These events were supported by logarithmic Bayes factors, computed from changes in single nucleotide polymorphism (SNP) density between the recombinant genome and its assigned lineage.

 In parallel, we used Gubbins [32] with default parameters to detect recombination events, both between MAH lineages and across different sources (e.g. humans, pigs and environmental samples), based on whole-genome alignments. Recombination hotspots were defined as regions with SNP densities in the top 0.1% within 1 kb windows, a threshold chosen to capture the most highly variable regions, as previously described [12]. SNP density was calculated per genome window size of 1 kbp, where each recombination event that occurred within the genome window was computed as an event per kilobase.

 For statistical analysis, we assessed data normality using the Shapiro–Wilk test. Depending on the outcome, either Pearson’s correlation coefficient or Spearman’s rank correlation coefficient was used to assess relationships between variables. Statistical significance was set at a *P* value of <0.05 and adjusted for multiple comparisons using Bonferroni’s correction method. Additionally, we calculated the recombination-to-mutation (r/m) ratio, which reflects the relative contribution of recombination versus mutation to genetic variation, and the ρ/θ ratio, representing the number of recombination events per vertically inherited SNP.

 For quality control, we compared the MAH genomes with 399 publicly available genomes of MAP (BioProjects: PRJNA306363, PRJNA739165 and PRJNA686527), a clonal subspecies known to undergo recombination at a much lower frequency [8,33, 34]. For the PRJNA686527 sequences, only one genome per animal was retained. Differences in recombination metrics between the MAH and MAP datasets were assessed using the Mann–Whitney *U* test, with statistical significance defined at *P*<0.05.

## Results

### Population structure of MAH identifies three novel lineages

 Of the 726 publicly available MAH genomes analysed, 702 passed quality control and were included in the downstream analysis. The total contig sizes of these assemblies ranged from 4,776,238 to 6,896,945 bp, with G+C content ranging between 66.72 and 69.41 mol% (Table S1). Sample origin information indicated that 313 were from Germany, 140 from Japan, 122 from the USA, 57 from Portugal, 55 from Switzerland and 15 from France. Regarding source, 643 samples originated from humans, while 30 and 29 were from pigs and water, respectively.

**Fig. 1.**
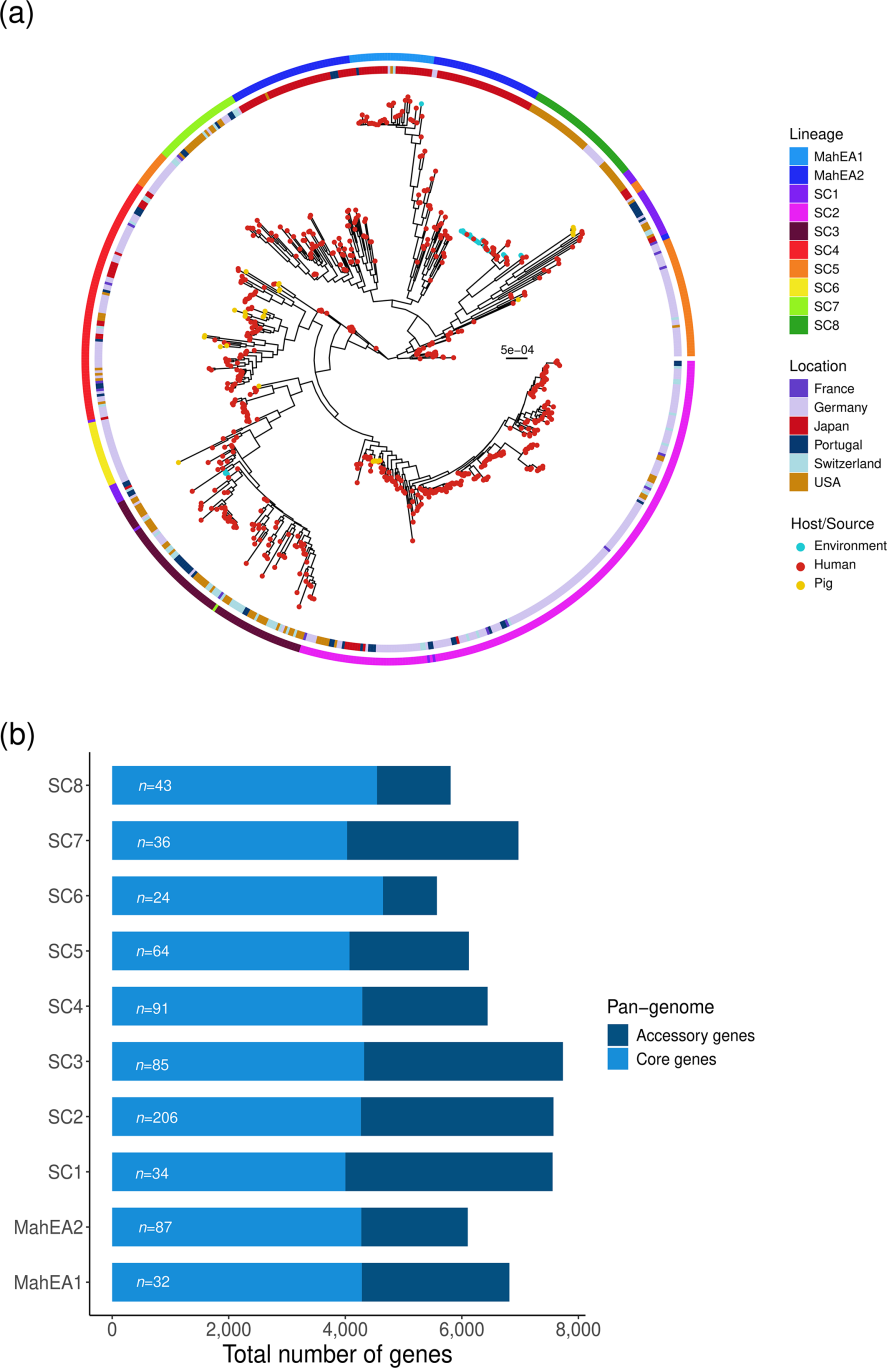
Population structure of 702 global MAH genomes. (**a**) Midpoint-rooted maximum-likelihood phylogenetic tree from core genes inferred with 236,698 single-nucleotide polymorphisms showing tips coloured according to host/source. The inner ring indicates the geographic origin of each MAH genome, while the outer ring represents the Bayesian population structure level inferred by rheirBAPS. (**b**) The total number of core and accessory genes associated with individual inferred lineages from pan-genome analysis.

 Phylogenetic reconstruction and Bayesian population structure analysis based on core-genome alignments revealed ten distinct MAH lineages (Lineages 1–10), seven of which corresponded to previously described groups. We identified three novel lineages, which we propose to name SC6, SC7 and SC8 (Table 1). Furthermore, our phylogenetic analysis suggests geographic associations for some of these lineages. As previously reported, MahEA1 and MahEA2 were predominantly composed of samples from Japan. Similarly, SC2, SC5 and SC6 were largely associated with European countries, while SC8 primarily included genomes from the USA and a few samples from Germany (Fig. 1a). All pig-derived MAH genomes originated from Japan, with an average of 16,987 SNPs (SD 5,620) separating pig and human genomes. Most pig genomes clustered within SC4 and were interspersed among human-derived samples from Europe and the USA. This pattern suggested phylogenetic association across hosts and geographic regions despite large genetic distances.

**Table 1.** Summary of the Bayesian population structure inferred in this study compared with nomenclature used in previous studies

Current population structure	Reference
MahEA1	Yano *et al*. [12]
MahEA2	Yano *et al*. [12]
SC1	Yano *et al*. [12]
SC2	Yano *et al*. [12]
SC3	Yano *et al*. [12]
SC4	Yano *et al*. [14]
SC5	Komatsu *et al*. [17]
SC6	This study
SC7	This study
SC8	This study

 We next examined genomic diversity across lineages. The mean number of core genes per lineage was 4,274 (sd, 208), with the fewest in SC1 and the most in SC6; however, the core genes of all genomes analysed together was 3,976. Conversely, SC1 exhibited the largest accessory genome, while SC6 had the smallest (Fig. 1b). The mean number of accessory genes across lineages was 2,395 (sd, 911), reflecting substantial variability in non-core content. Finally, we observed differences in predicted plasmid content: SC6 had the fewest plasmids per genome, with a mean (sd) of 0.83 (0.38), whereas SC8 had the highest, with 2.30 (0.91) plasmids per genome (Fig. S1). This variation in plasmid content reflects broader divergence in accessory genome composition, which may relate to ecological or functional characteristics across lineages.

### Homologous recombination drives evolution in MAH genomes

**Fig. 2.**
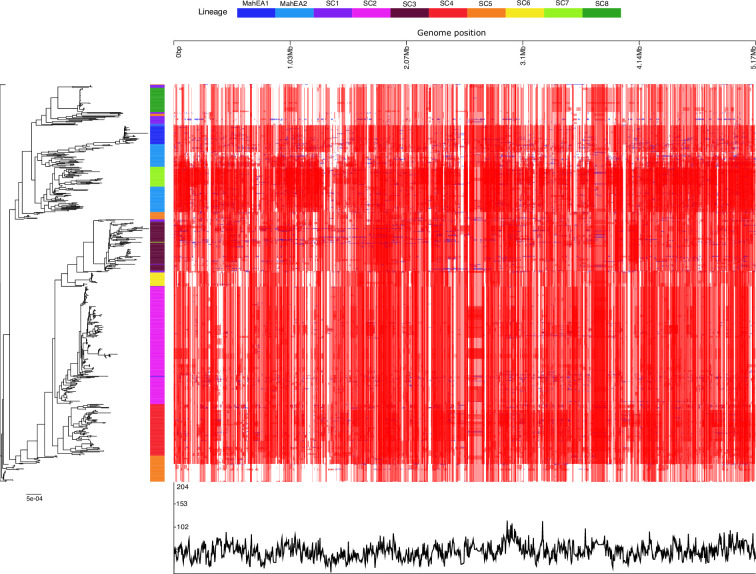
Maximum-likelihood phylogeny generated from non-recombinant regions of the whole-genome alignment of 702 MAH isolates, with the updated population structure (MahEA1, MahEA2, and SC1 to SC8). The top horizontal bar indicates the genomic coordinates of the NZ_CP018019.2 reference genome. In the heatmap, the red bars depict recombination events that occur on the internal nodes of the tree corresponding to inheritance of multiple descendant isolates, while the blue bars indicate recombination events on the terminal branches of the tree, correlating to a single genome. The line graph at the bottom represents the distribution of recombination events across the genome from all MAH lineages.

**Fig. 3.**
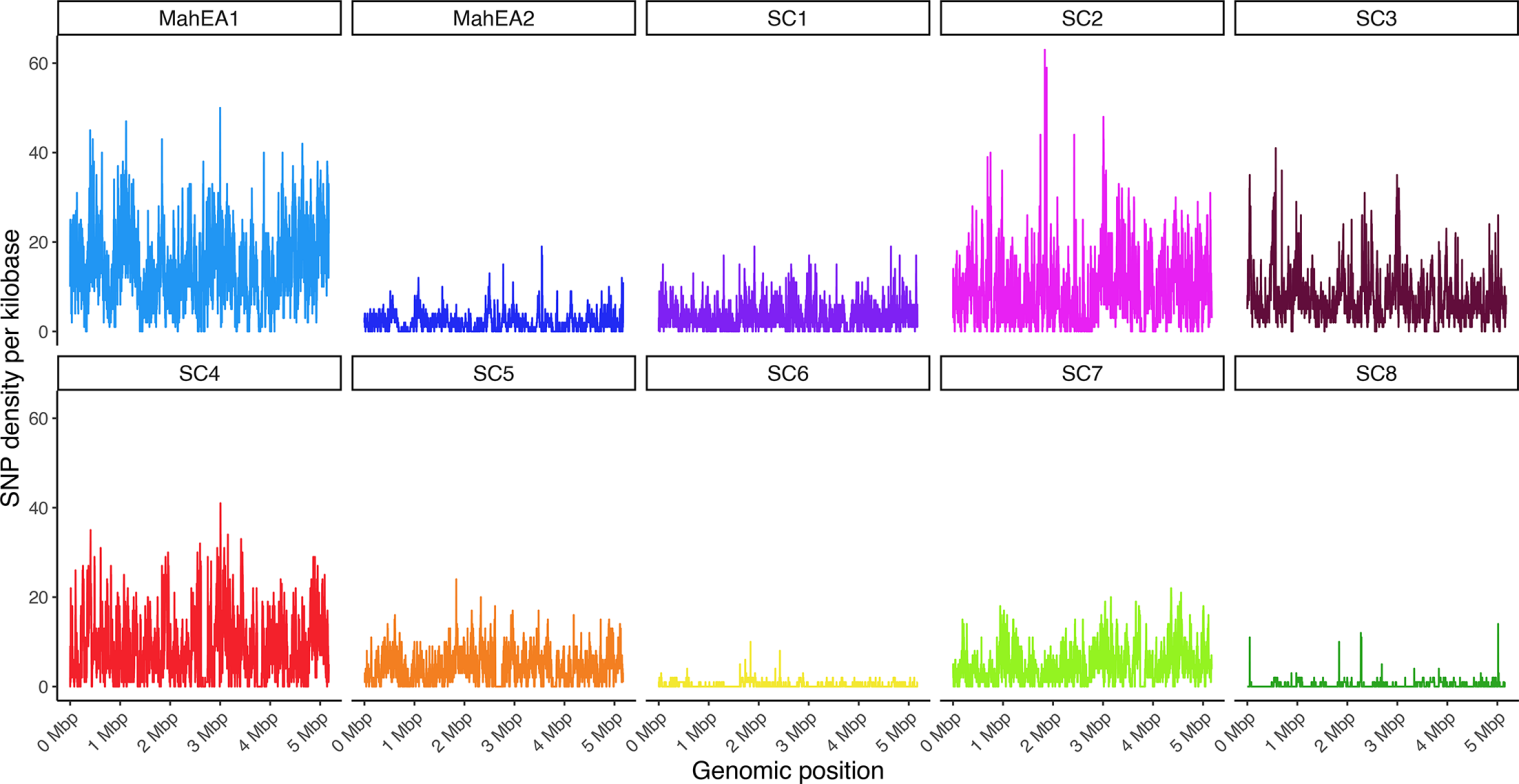
Recombination events detected in MAH genomes, grouped by lineage.

 Recombination analysis of the full set of 702 MAH genomes revealed extensive polymorphism distributed throughout the genome, which could be related to homologous recombination or vertically inherited SNPs (Fig. 2). To better understand recombination within the population structure, we evaluated genetic exchange between genomes within each lineage. MahEA1, SC2, SC3, SC4 and SC7 showed high recombination rates, with mean (sd) SNP densities of 14.07 (7.96), 8.39 (7.05), 7.36 (5.17), 8.13 (6.69) and 4.53 (3.72) SNPs per kilobase, respectively. In contrast, MahEA2, SC6 and SC8 showed substantially lower recombination, with mean SNP densities of 1.97 (1.90), 0.25 (0.53) and 0.35 (0.78) per kilobase, respectively (Fig. 3).

 To differentiate the contributions of homologous recombination and vertical inheritance to genetic diversity, we compared the r/m and ρ/θ ratios among MAH lineages, and also with those of MAP genomes from isolates collected in Canada, Ireland and the USA (Fig. S2). Our results indicate that genetic diversity in MAH is primarily driven by homologous recombination, which contributed at least twice as much as vertically inherited SNPs (r/m, 1.97), compared with MAP, which had a much lower r/m ratio of 0.05 (Table 2). Interestingly, our results indicate that the SC1 genetic diversity was most likely driven by vertically inherited SNPs, while both recombination and vertical inheritance contributed comparably to the diversity observed in SC6. To further contextualize these findings, we compared SNP density between MAH and MAP genomes. As expected, we observed significantly lower SNP density in the MAP genomes, with a mean (sd) of 0.02 (0.21) SNPs per kilobase, compared with 59.33 (19.82) SNPs per kilobase in MAH (Mann–Whitney *U*=24,198,766; *n*_1_=5,173; *n*_2_=4,678; two-sided; *P*<2.2×10^−16^) (Fig. S3A). Despite analysing unique genomes from three different countries, our results show that MAH is more diverse than MAP, consistent with findings from previous studies [8,33, 34]. This could be due to the wide range of ecological niches that MAH can adapt to and its genetic plasticity, unlike MAP, which has a narrower host range. Pairwise ANI heatmap plots of genomes in our study corroborate with these reports that MAP is considered less diverse than MAH (Fig. S3B).

**Table 2.** Homologous recombination metrics reflecting genetic diversity in selected *M. avium* subsp. and lineages of MAH

*M. avium* subsp.	Average no. of recombination block	R/m ratio	No. of homologous recombination events per vertically inherited SNP (ρ/θ)
MAH	33.80	1.97	0.06
MAP	0.10	0.05	0.01
**MAH lineage**			
MahEA1	76.14	2.08	0.06
MahEA2	17.83	23.42	0.37
SC1	57.96	0.29	0.01
SC2	16.61	10.57	0.31
SC3	26.02	12.09	0.21
SC4	36.49	2.33	0.07
SC5	27.02	1.47	0.04
SC6	8.04	1.09	0.04
SC7	59.06	1.39	0.04
SC8	2.41	8.67	0.13

**Fig. 4.**
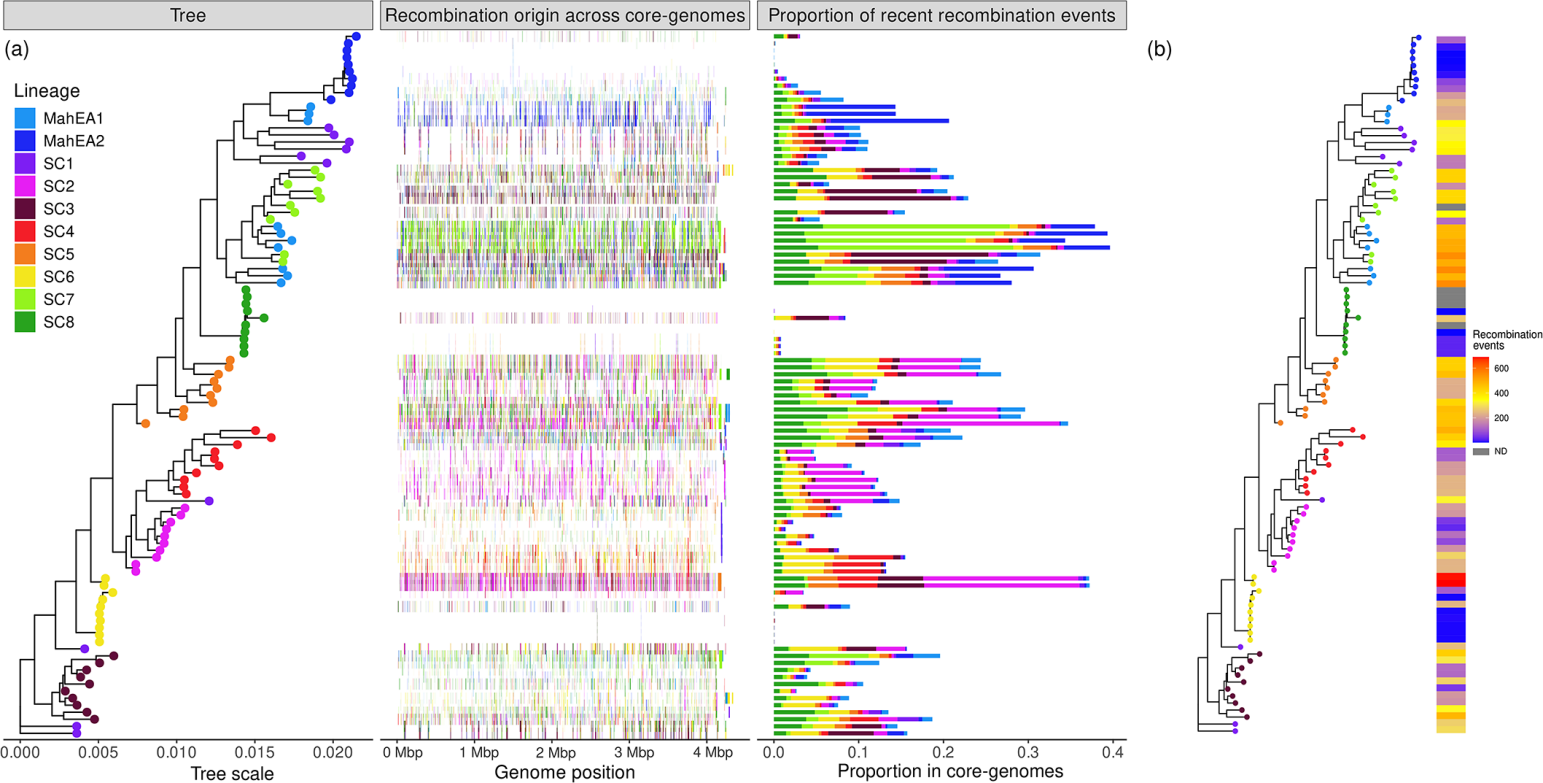
Inferred recombination events among a subsample (*n*=100) of MAH genomes representing all lineages. (**a**) Left panel: Maximum-likelihood phylogenetic tree with tips coloured according to lineage. Middle panel: Origins of recent horizontal recombination events detected by fastGEAR, with colours indicating the contributing lineages detected in this study. Right panel: Proportion of detected recent recombination events in the core genome, with colours indicating donor lineages. (**b**) Maximum-likelihood phylogenetic tree with tips coloured according to lineage, with heatmap illustrating the number of recombination events. ND: none detected

 Using the inferred population structure from rhierBAPS, we identified possible recombination events between lineages and quantified gene exchange events across genomes. In our subsampled dataset, we observed extensive inter-lineage recombination, with fragments of a median length of 1.5 kbp shared across the core genomes (Fig. 4). However, the extent of recombination varied by lineage. For example, SC8 exhibited limited gene exchange, with recombination fragments from other lineages accounting for less than 10% of the core genome. By comparison, four genomes of MahEA1 had up to 40% of their core genome from donor sequence fragments originating from other lineages. Notably, most of the gene exchange in MahEA1 was attributed to donor fragments from MahEA2 and SC7, which together accounted for nearly half of its inter-lineage recombination events (Fig. 4).

 To assess whether the observed inter-lineage recombination might be associated with genome fragmentation or assembly artefacts, we examined the relationship between the number of contigs and inter-lineage recombination events. A Spearman’s rank correlation analysis yielded a correlation coefficient of 0.26 and a *P* value of 0.011, indicating a weak but statistically significant positive relationship, potentially reflecting the influence of assembly fragmentation (greater contig counts) on recombination detection (Fig. S4).

### Genes affiliated with recombination hotspots in MAH

**Fig. 5.**
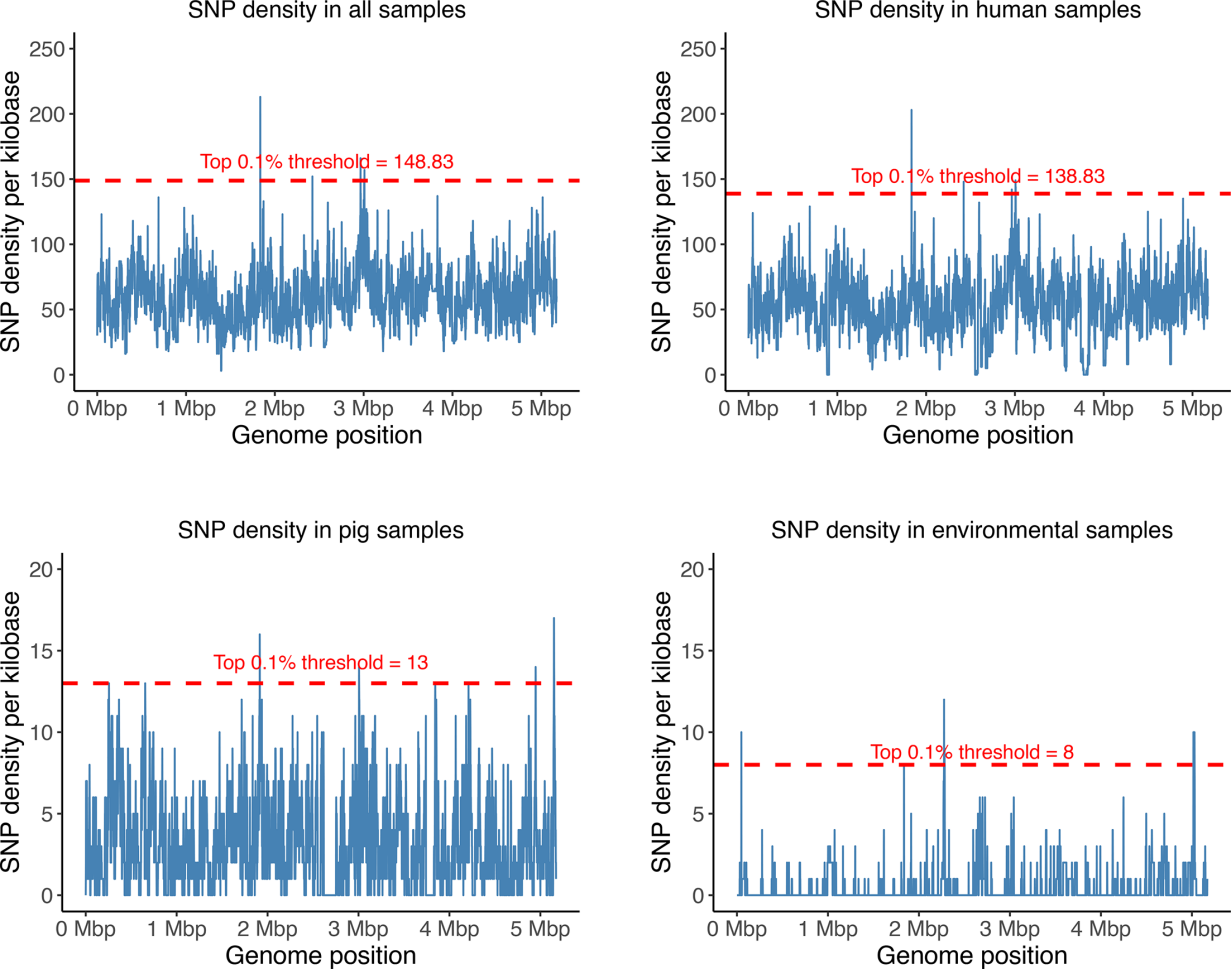
Recombination events identified in MAH genomes grouped by host/source. The horizontal dotted red line highlights recombination hotspots.

 To identify genomic regions potentially under selective pressure across different niches, we focused on regions with the highest recombination rates (top 0.1% of all recombination events). When all MAH genomes were analysed collectively, we identified four high-frequency recombination hotspot regions. The same hotspots were also detected when human-derived MAH genomes were analysed separately. However, analyses of MAH genomes originating from pigs and the environment revealed additional hotspots, with eight and nine regions identified, respectively (Fig. 5).

 The most prominent hotspot across the full dataset was an ATP-binding cassette (ABC) domain-containing protein, which is part of the *marR* operon, with 213 recombination events per kilobase. ABC transporters are membrane proteins that facilitate the transport of a variety of substrates, and the *marR* operon is known to regulate responses to environmental stressors and facilitate antimicrobial resistance [35]. Upon closer inspection of the ABC domain-containing protein, we found that it was flanked by two tRNAs, one located 35 kbp upstream and the other 16 kbp downstream. This hotspot was consistently detected in the human and environmental genomes but not found in pig-associated MAH genomes (Table 3).

**Table 3.** Summary of recombination hotspots in MAH, grouped source of collection Genome coordinates represent regions in the NZ_CP018019.2 reference genome.

Genome source	Gene	Genome coordinate
All genomes	ABC domain-containing protein^*^	1836706–1836715
	Intergenic region between CaiB/BaiF CoA-transferase family protein and UPP	2423114–2423121
	NRPSs	2964997–2967570
	MmpL4 transporter	3006201–3009988
	MmpL4 transporter†	3007975–3009988
Humans	ABC domain-containing protein^*^	1836706–1836715
	Intergenic region between CaiB/BaiF CoA-transferase family protein and UPP	2423114–2423121
	NRPSs	2964997–2967570
	MmpL4 transporter†	3006088–3009994
Pigs	Class I SAM-dependent methyltransferase	255943–256887
	CBP family intramembrane glutamic endopeptidase and sterol-carrier family protein	654010–654492
	Carboxymuconolactone decarboxylase family protein	1914400–1914906
	MmpL4 transporter†	3006468–3007254
	DUF448 domain-containing protein^‡^	3844181–3844438
	HNH endonuclease signature motif-containing protein	4209419–4210816
	YhgE/Pip domain-containing protein^§^	4948822–4948922
	Alpha/beta hydrolase and hypothetical protein	5149992–5151764
Environment	DUF5632 domain-containing protein^‡^	48310–48320
	ABC domain-containing protein^*^	1836706–1836715
	ACR3 family arsenite efflux transporter	2274707–2276214
	Arsenite methyltransferase	2276316–2277116
	TniQ family protein	2280804–2281715
	ATP-binding protein	2281705–2282730
	Mu transposase C-terminal domain-containing protein	2282718–2284793
	TetR family transcriptional regulator	5015439–5016086
	Maltokinase N-terminal cap-like domain-containing protein	5027797–5029146

*tRNA both downstream and upstream.

†Next to an insertion sequence.

‡tRNA downstream or upstream.

§Next to a virulence factor.

CBP, CREB-binding protein; CoA, coenzyme A; HNH, histidine–asparagine–histidine; SAM, S-adenosylmethionine; TetR, tetracycline repressor; UPP, undecaprenyl-diphosphate phosphate.

 Other notable recombination hotspots spanned larger genomic regions, including a non-ribosomal peptide synthetase (NRPS) gene and a resistance–nodulation–division family transporter (MmpL4 transporter), covering 2.5 and 3.7 kbp of the genome, respectively, with 166 and 157 recombination events per kilobase. NRPS genes are involved in the synthesis of secondary metabolites, which can play roles in virulence and environmental adaptation, while MmpL4 is often associated with iron acquisition and virulence in mycobacteria [36]. These regions may be subject to selective pressure, and the proximity of the MmpL4 transporter to an IS630 transposase suggests potential mobility or horizontal gene transfer.

 For isolates collected from pigs, the largest recombination hotspot spanned a 1.7 kbp region in the alpha/beta hydrolase – enzymes often involved in diverse metabolic processes – and a hypothetical protein of unknown function, as well as the MmpL4 transporter also found in human isolates. Additional hotspots included a DUF488 domain-containing protein positioned downstream of a tRNA, a carboxymuconolactone decarboxylase family protein involved in aromatic compound degradation and a YhgE/Pip domain-containing protein positioned upstream of virulence-associated Mammalian cell entry (Mce) family proteins. Surprisingly, we observed fewer recombination events in MAH genomes from environmental sources compared with those from human or pig origins. The largest hotspot was a 1.7 kbp region in an ATP-binding protein and a Mu transposase C-terminal domain-containing protein, which may contribute to genome plasticity. Other hotspots included a DUF5632 domain-containing protein downstream of a tRNA, an ABC domain-containing protein and a TniQ family protein, known to mediate the transposition of the mercury-resistance transposon Tn5053 and the dissemination of integrons [37].

 To determine whether recombination events identified by Gubbins were consistent with inter-lineage recombination detected by fastGEAR, we further analysed the MmpL4 transporter in all MAH genomes. Recombination was observed across nearly the entire gene, with a 1.5 kbp region (spanning the first half of the gene) likely originating from SC7 genomes (Fig. S5).

## Discussion

 In this study, we used publicly available genomic data to expand the current understanding of MAH population structure. In addition to confirming the major lineages described in previous work [12,14, 17], we identified three additional lineages: SC6, SC7 and SC8. By analysing a large dataset of 702 isolates, we were able to detect patterns of geographic and host associations across lineages, such as SC2, SC5 and SC6, that primarily consist of genomes from Europe, suggesting potential regional restriction.

 Although plasmid content varied between MAH lineages, no clear association was observed with geographic location or host origin. Notably, SC8 had the highest mean of plasmids per genome and included sequences from both human and environmental sources. This pattern may be indicative of increased horizontal gene transfer in environmental contexts, where diverse NTM species and other MAH strains coexist and interact. This observation aligns with a previous report showing that environmental MAH strains are more likely to serve as conjugative donors than clinical strains [38].

 MAH is known to be highly diverse and to undergo frequent homologous recombination, which could possibly generate new variants through admixture of donor lineages in shared environments [12,17]. Contrary to earlier reports that described SC5 as minimally recombinant [17], we observed frequent recent inter-lineage recombination involving this lineage. This discrepancy may be due to the inclusion of a diverse collection of European MAH genomes in our analysis, compared with earlier datasets, which primarily comprised more genetically uniform pig-derived samples from Japan. Furthermore, some members of MahEA1 had larger donor fragments from MahEA2, consistent with previous findings [12,17], likely related to their shared geographic origin. However, we also identified MahEA1 genomes with more donor fragments from SC2 and SC7, representing a European cluster and one of the novel but geographically disperse lineages, highlighting the genetic heterogeneity within MahEA1, as previously reported [14]. A recent study that compared the recombination rate across different bacteria genera saw that *M. avium* had the highest r/m (5.44) among the species examined (*M. avium*, *Mycobacterium fortuitum*, *Mycobacterium intracellulare*, *Mycobacterium kansasii*, *M. tuberculosis*, *Mycobacterium abscessus* and *Mycobacterium chelonae*), with *M. tuberculosis* having the least (0.37) [39]. The recombination rate of *Mtb* is known to be low, as there is little evidence of horizontal gene transfer, unlike MAH and other NTM [40,41]. However, our inter-species comparison of r/m shows that MAH undergoes higher recombination than MAP, which is a known clonal subspecies of *M. avium* [8,42].

 Through examination of recombination hotspots across the genomes, we identified regions that are essential for MAH survival, virulence and antimicrobial resistance, such as ABC proteins and the MmpL4 transporter [43,44]. These large membrane proteins act as transmembrane or efflux transporters for the extrusion of antibiotics and peptides in mycobacteria and other bacteria, while ABC proteins can either serve as importers or exporters [45,46]. In our analysis, the ABC transporter was found within the *marR* (multiple-antibiotic resistance regulator) operon, which has been shown to increase rifampicin sensitivity, though *marR* itself can regulate efflux and contribute to rifampicin resistance in *Mycobacterium smegmatis* [47].

 Notably, the MmpL4 transporter was located upstream of an IS630 transposase, suggesting possible mobilization via transposable elements. Similarly, the ABC transporter genes were flanked by tRNAs, known hotspots for mobile genetic elements and integrative and conjugative elements [48,50]. The MAH NZ_CP018019.2 reference genome contains 47 tRNA genes, distributed across the genome, and their role in facilitating recombination has been understudied. They are likely to play a key role in MAH recombination as they are targets for integrating foreign DNA and have been horizontally transferred by phages in *Mycobacterium mucogenicum* and *Mycobacterium neoarum*, underscoring their importance in the genomic evolution of mycobacteria [51,52]. A critical examination of the MmpL4 transporter revealed that it was highly recombinant, and knockout experiments showed that both MmpL4 and MmpL5 are crucial for virulence in mycobacteria [53,54].

 Another recombination hotspot we identified was an NRPS, which contributes to cell wall integrity and immune evasion, potentially making it a target of selective pressure [55]. We also found domains of unknown function (DUF) genes, such as DUF488 and DUF5632, downstream of tRNA genes in environmental and pig-derived isolates. The significance of these DUFs remains unclear but warrants further investigation.

 One unexpected finding was the lower recombination rate among environmental isolates, which contrasts with the expectation that recombination would be more frequent in complex microbial communities. This may reflect limitations of our study, including sampling bias and the disproportionately low number of MAH genomes from environmental sources in our dataset. However, given that MAH is an opportunistic pathogen likely acquired from the environment, the recombination events observed in human-derived isolates may have occurred prior to infection. Another limitation is the use of short-read sequences, which can lead to genome fragmentation and potentially inflate the apparent number of recombination events. Although we observed a high frequency of inter-lineage recombination overall, our statistical analysis found a weak positive correlation between the number of recombination events and contigs per genome. This suggests that while Illumina-based assembly quality may contribute to the appearance of mosaicism, it is not likely to be the primary contributor to the observed recombination signal. Future studies should aim to reduce this bias by employing hybrid assembly approaches that combine short- and long-read sequencing to improve genome assembly quality and the accuracy of recombination detection.

Overall, this study provides an updated view of the MAH population structure, offers new insights into its evolutionary dynamics and identifies genomic regions frequently affected by homologous recombination. By expanding our understanding of MAH genomic diversity, this work lays the foundation for improved lineage-specific genotyping tools and further investigation into virulence factors and antimicrobial resistance determinants to support One Health surveillance efforts.

## Supplementary material

10.1099/mgen.0.001543 Uncited Supplementary Material 1.

10.1099/mgen.0.001543 Uncited Table S1.

## Abbreviations

ABC: ATP-binding cassette
ANI: average nucleotide identity
DUF: domains of unknown function
MAH: *Mycobacterium avium* subsp. *hominissuis*
MahEA1: MahEastAsia1
MahEA2: MahEastAsia2
MAP: *Mycobacterium avium* subsp. *paratuberculosis*
NRPS: non-ribosomal peptide synthetase
NRPS: non-ribosomal peptide synthetase
NTM: non-tuberculous mycobacteria
r/m: recombination-to-mutation
SC: sequence cluster
SRA: Sequence Read Archive
