# Supplementary material for: Population structure of Mycobacterium avium subsp. hominissuis provides new insights into genomic evolution
Source: Microb Genom. 2025 Nov 4;11(11):001543. doi: 10.1099/mgen.0.001543 (PMC12584985; doi:10.1099/mgen.0.001543)
Supplement: Uncited Supplementary Material 1. [file mgen-11-01543-s001.pdf]

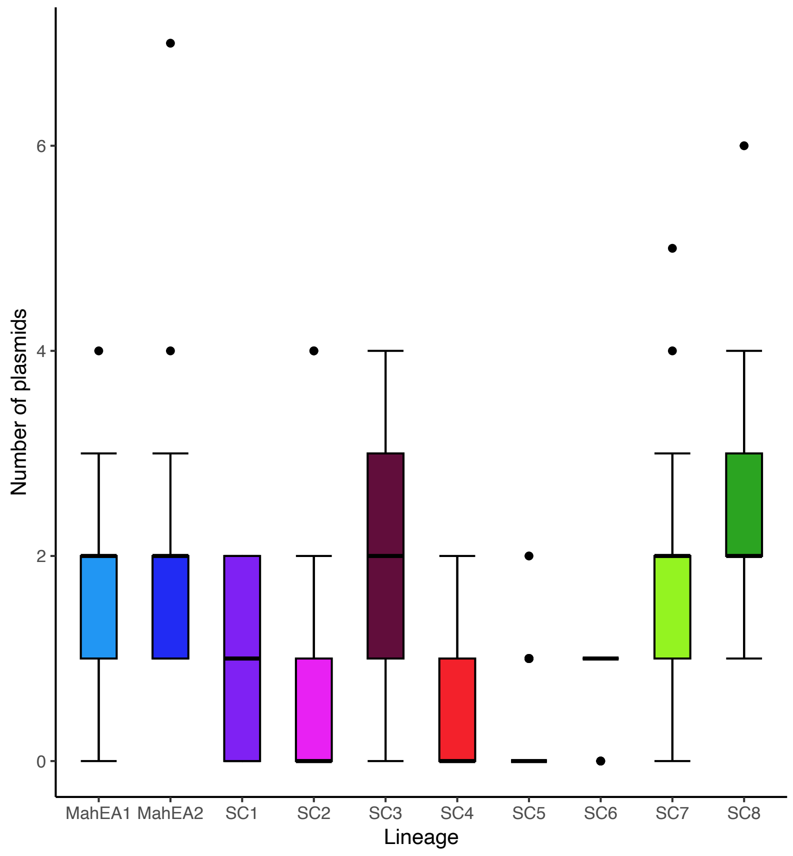

Supplementary Figure S1. Plasmid detection across different lineages of *Mycobacterium avium* subsp. *hominissuis* with MOBsuite.

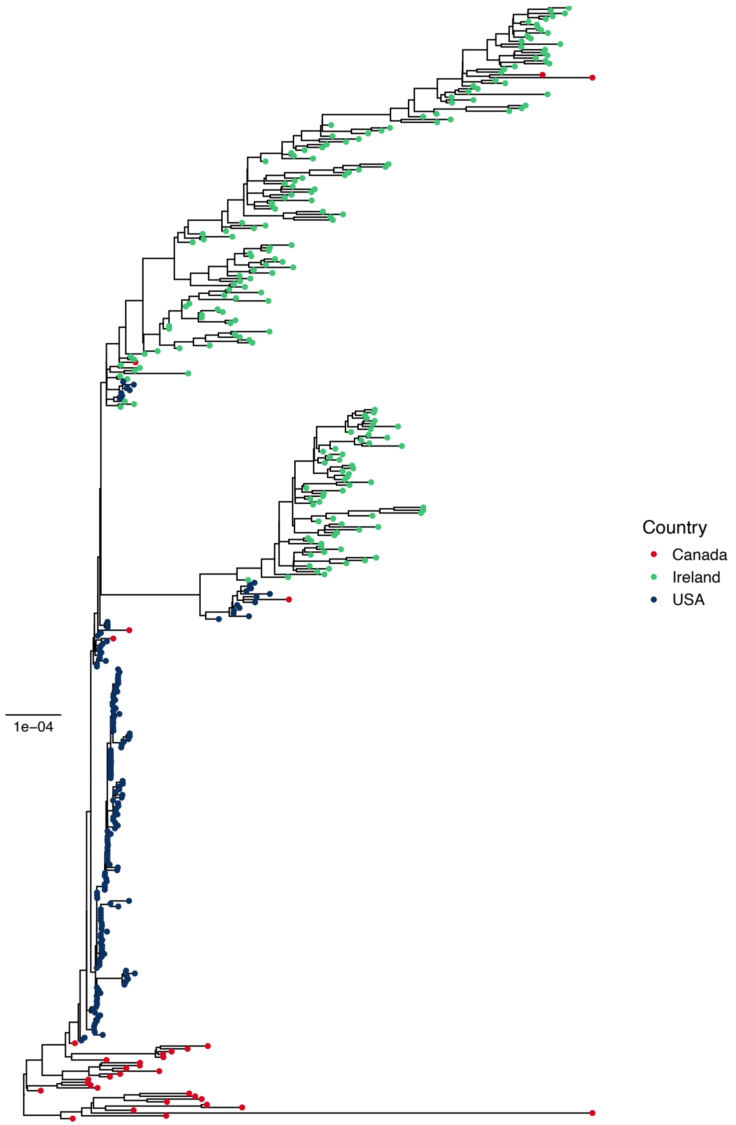

Supplementary Figure S2. Maximum likelihood phylogenetic tree of 399 isolates of *Mycobacterium avium* subsp. *paratuberculosis* constructed from core genes inferred with 28,085 single-nucleotide polymorphisms. Tree tips are coloured according to the country sources of the isolates.

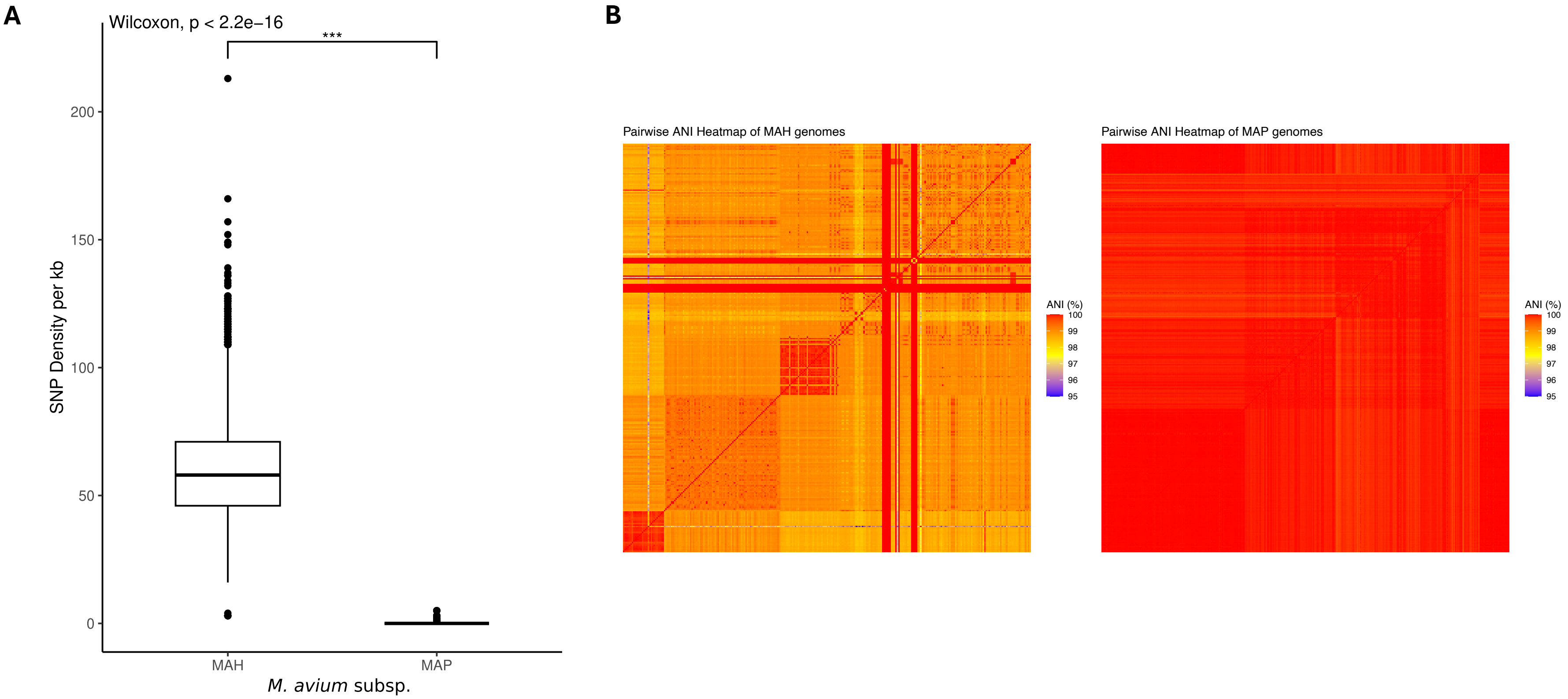

Supplementary Figure S3. Genomic diversity of *M. avium* subspecies. **A)** Boxplot of SNP densities per kilobase across genomes of *Mycobacterium avium* subsp. *hominissuis* (MAH) (n = 702) and *M. avium* subsp. *paratuberculosis* (MAP) (n = 399). Statistical significance was assessed using the Mann-Whitney *U* test (Wilcoxon rank-sum), with a significance threshold of p-value < 0.05. **B)** Pairwise average nucleotide identity (ANI) heatmap of *Mycobacterium avium* subsp. *hominissuis* (MAH) (n = 702) and *Mycobacterium avium* subsp. *paratuberculosis* (MAP) (n = 399) genomes.

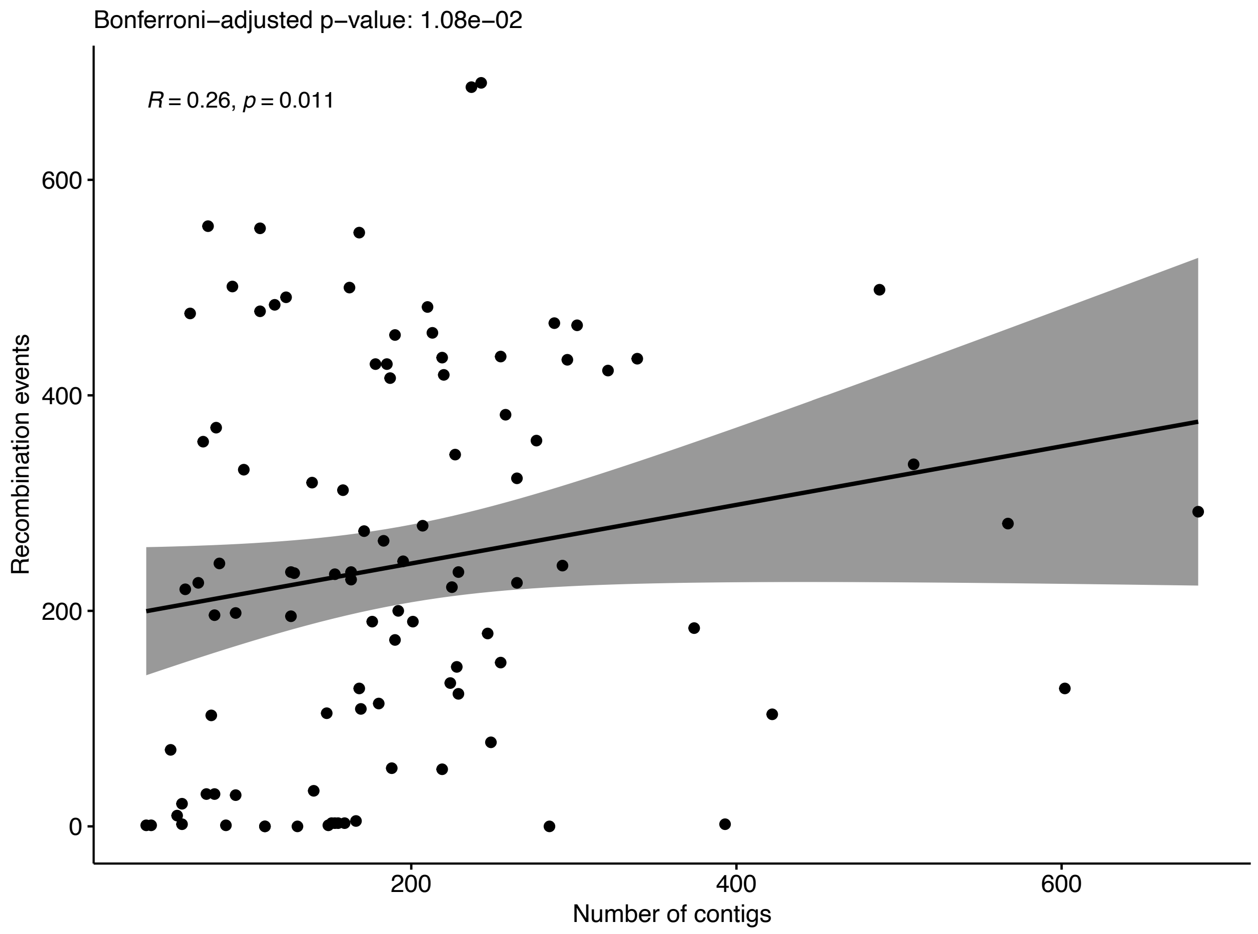

Supplementary Figure S4. Scatterplot of inter-lineage recombination events and number of contigs in the genome assembly. Spearman's rank correlation coefficient (*R*) between the two datasets was 0.26 with a Bonferroni adjusted p-value of 0.0108.

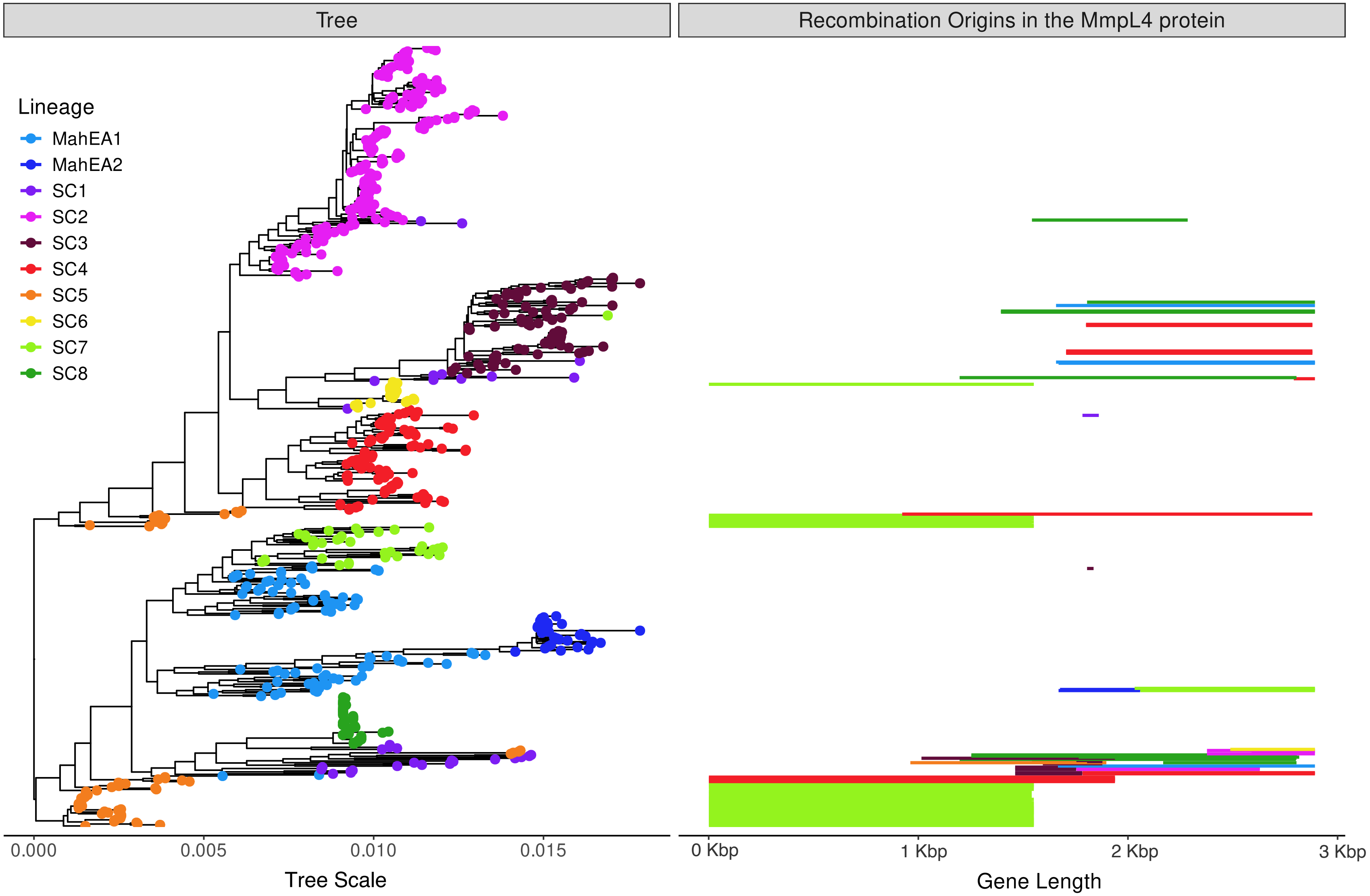

Supplementary Figure S5. Recombination blocks inferred by fastGEAR in the MmpL4 transporter region, identified as a hotspot among all 702 *Mycobacterium avium* subsp. *hominissuis* genomes.
